# Awareness, Attitudes, and Barriers to Breast Cancer Screening Among Breast Screening Health Education Week Attendees at King Saud Medical City, Riyadh

**DOI:** 10.1155/ijbc/7289188

**Published:** 2025-12-11

**Authors:** Atheer Alturki, AbdulRahman M. Elnasieh, Razan Alhadlaq, Firas Al Saluly, Faisal Bin Hawidi, Ahoud S. Alzuwaidi, Taghreed Mohammed Alaidarous, Khalid M. Ajarim, Meshari K. Alharabi

**Affiliations:** ^1^ King Saud Medical City, Riyadh, Saudi Arabia, ksmc.med.sa; ^2^ Preventive Medicine and Public Health at King Saud Medical City, Riyadh, Saudi Arabia

**Keywords:** attitudes, awareness, barriers, breast cancer screening, Saudi Arabia

## Abstract

**Background:**

Breast cancer is among the leading causes of mortality in women globally, with a rising incidence in developing countries, including Saudi Arabia. Early detection through screening is essential for reducing the mortality rate among women. However, factors such as awareness, attitudes, and barriers significantly influence women′s participation in screening programs, especially in resource‐limited settings.

**The Aim of the Study:**

The aim of this study is to assess the awareness, attitudes, and barriers to breast cancer screening among breast cancer screening health education week attendees at King Saud Medical City, Riyadh.

**Materials and Methods:**

The study was conducted during breast cancer screening health education week at King Saud Medical City in Riyadh, from October 13 to 17, 2024. A convenience sample of 277 women, aged 18–60, completed a structured, self‐administered questionnaire. Data on awareness, attitudes, and barriers related to breast cancer screening were analyzed using descriptive and inferential statistics in SPSS Version 23.0.

**Results:**

Among the women surveyed, 63.5% showed a good level of awareness about breast cancer screening, with an average awareness score of 12.6 ± 5.5 out of 23. Positive attitudes toward screening were common, with 97.8% expressing supportive views (*p* = 0.045). Higher awareness levels were significantly associated with age (*p* = 0.003) and occupation (*p* = 0.001), while attitudes toward screening were significantly linked to education. The main barriers reported were lack of time (30.3%), difficulty accessing services (15.2%), and fear of the procedure (15.2%). In the multiple logistic regression analysis, any significant predictors of awareness and attitude toward breast cancer screening were not identified.

**Conclusion:**

The study indicates a relatively high level of awareness and a positive attitude toward breast cancer screening among participants. However, significant barriers, including time constraints, accessibility challenges, and fear of the procedure, limit regular participation. To increase screening rates and reduce breast cancer mortality in Saudi Arabia, it is recommended to implement targeted awareness campaigns, enhance service accessibility, and provide continuous education through healthcare providers.

## 1. Introduction

Breast cancer ranks as the most common malignancy affecting women globally and remains the leading cause of cancer‐related mortality among this demographic [1]. In 2022 alone, approximately 2.3 million women were diagnosed with breast cancer, and 670,000 women died from the disease, making it the single largest contributor to cancer‐related deaths in women worldwide [2]. Although historically more prevalent in developed countries, incidence rates have risen sharply in developing nations over the past two decades. Global estimates underscore stark disparities in burden: in high human development index (HDI) nations, the lifetime diagnosis rate is 1 in 12 women, with a mortality rate of 1 in 71. In contrast, in low‐HDI nations, the diagnosis rate is lower at 1 in 27, yet the mortality rate is substantially higher at 1 in 48, reflecting inequalities in healthcare access, awareness, and early detection efforts [2].

Breast cancer is a multifactorial disease shaped by biological, genetic, and lifestyle determinants. Risk factors include age, hormonal changes, family history, obesity, insufficient physical activity, genetic mutations such as BRCA1/2, and lifestyle‐related elements including smoking and diet [3]. Although once considered more common in Western countries, rising incidence in Asian and Middle Eastern nations has been reported, accompanied by comparatively higher mortality rates due to later‐stage diagnosis and limited awareness [4].

### 1.1. Breast Cancer in Saudi Arabia

In Saudi Arabia, breast cancer is the most frequently diagnosed cancer among women and constitutes a substantial proportion of the national cancer burden. According to the Ministry of Health, of the 2741 new cancer cases recently recorded, approximately 19% were breast cancer [5]. National data from the Saudi Cancer Registry reported that breast cancer accounted for 28.7% of all cancer cases among women [6]. Additionally, breast cancer contributes to an estimated 13.08% of all cancer‐related deaths in the Kingdom [7].

Lifestyle changes, rising obesity rates, advancing maternal age, family history, and reduced physical activity are prominent contributors to the growing prevalence in Saudi women. Importantly, compared to Western countries, breast cancer in Saudi Arabia is frequently diagnosed at younger ages, often in premenopausal women and at more advanced stages of disease, further compounding morbidity and mortality [7–9].

### 1.2. Advances in Detection and Screening

Improvements in imaging technologies and the establishment of screening programs have enhanced early detection capabilities worldwide. Clinical breast examinations (CBEs), breast self‐examinations (BSEs), and mammography are the most commonly promoted methods, with mammography recognized as the gold standard. Evidence demonstrates that mammography reduces breast cancer mortality by 20%–40% among women aged 50–69 years [10] and contributes to up to a 23% reduction in breast cancer deaths when implemented systematically [11]. Early detection enables therapeutic interventions at more treatable stages, improving survival rates and reducing treatment complexity [12].

The importance of healthcare professionals in disseminating accurate information, fostering trust through effective doctor–patient relationships, and encouraging adherence to screening cannot be overstated. Studies highlight that informed and supportive physician–patient interactions improve participation in screening programs and enhance women′s confidence in preventive health behaviors [13, 14].

### 1.3. Breast Cancer Screening in Saudi Arabia

Saudi Arabia has taken important steps toward improving early detection. Mammography was first introduced in 2002, with free national screening services later provided by the Ministry of Health. A nationwide breast cancer screening program was established in 2007, initially screening 1215 women in its first year [15]. Regional initiatives, such as the Al Qasim program targeting women aged 35–60, were launched with accompanying awareness campaigns to improve uptake. By 2005, mass screening centers and organized national programs became accessible across all regions of the Kingdom.

Despite this progress, uptake of screening remains markedly low. The 2015 National Saudi Health Interview Survey revealed a screening participation rate of only 8% among eligible women [15]. This figure contrasts sharply with international benchmarks: breast cancer screening rates in the United Kingdom reached 70% in 2023–2024 [16], while in the United States, the rate was 79.8% in 2023 [17]. The disparity highlights critical challenges within Saudi Arabia, where available services are substantially underutilized, leading to delayed diagnoses and poorer prognoses [9].

Multiple studies have attributed underutilization to a range of factors, including limited awareness of screening guidelines, cultural stigmas, fear of the procedure, embarrassment, and logistical barriers such as distance to facilities or lack of time [8, 18]. These barriers echo patterns observed in other developing nations and underline the need for context‐specific strategies that address cultural sensitivities and structural limitations.

### 1.4. Rationale for the Present Study

The persistent underutilization of breast cancer screening in Saudi Arabia despite free nationwide availability raises urgent concerns. Previous studies across different regions of the Kingdom consistently point to inadequate awareness, misconceptions about screening, and significant cultural and systemic barriers [8, 9, 13]. Moreover, the higher prevalence of advanced‐stage diagnoses and younger onset among Saudi women compared to Western counterparts accentuates the need for early detection initiatives tailored to the local population [8, 13].

This research is grounded in the necessity to better understand Saudi women′s awareness, attitudes, and barriers regarding breast cancer screening. By examining knowledge of methods such as mammography, CBE, and BSE, alongside attitudes toward participation and perceived obstacles, the study seeks to provide insights that can inform more effective educational campaigns, community engagement strategies, and healthcare system interventions. Ultimately, strengthening screening uptake aligns with Saudi Arabia′s broader Vision 2030 health goals, which emphasize preventive medicine, improved women′s health, and reductions in cancer‐related mortality.

### 1.5. Objective

Our objective is to examine the level of awareness, attitudes, and obstacles related to breast cancer screening, with the primary goal of emphasizing the urgent need for enhanced awareness and early detection initiatives.

## 2. Methodology

### 2.1. Study Design and Setting

This cross‐sectional research was carried out at King Saud Medical City (KSMC) in Riyadh during a health education campaign for breast cancer screening, which took place from October 13 to 17, 2024. The campaign aimed to educate women on the importance of regular screenings and self‐examinations to detect breast cancer at an early stage. The study targeted the general population attending the education campaign. The attendees selected for the breast cancer screening education campaign at KSMC in Riyadh were chosen based on their potential impact in spreading awareness about the importance of early detection initiatives. By targeting a diverse group from the general population, we aimed to reach individuals who could benefit from learning more about regular screenings and self‐examinations for breast cancer. This strategic selection process was crucial in ensuring that the urgent message of enhanced awareness and early detection was effectively communicated to those attending the campaign.

### 2.2. Study Population

The study targeted women aged 20 and older who participated in the event. Informed consent was taken before the inclusion of participants. Voluntary participation was mandatory. Every attendee was asked about the history of breast cancer or currently receiving treatment before the dissemination of the study questionnaire. Women with a prior history of breast cancer or those currently receiving treatment were not included in the study.

### 2.3. Sampling Technique

The study employed a convenience sampling method, whereby all women who attended the health education campaign and consented to participate during the data collection phase were included. This nonprobability approach was chosen due to its practicality and feasibility in reaching the target population within the limited timeframe of the campaign. Convenience sampling is commonly used in community‐based health studies, particularly when access to the entire population is not possible [19].

### 2.4. Data Collection Tool

Data were gathered through a self‐administered, predesigned, and pretested questionnaire. The questionnaire used in this study was adapted from previously validated instruments, and prior to data collection, it underwent a pilot test with 30 participants from the target population to ensure clarity and cultural appropriateness. Although internal consistency statistics (e.g., Cronbach′s alpha) were not calculated, the instrument demonstrated acceptable reliability during the pilot phase, with participants reporting that items were understandable and relevant. To ensure cultural suitability in the Saudi context, the questionnaire was translated into Arabic using a forward–backward translation process and reviewed by a bilingual expert panel consisting of oncologists, public health specialists, and social scientists. Minor modifications were made to adapt certain items to local cultural sensitivities, particularly regarding BSE and screening practices. Content validity was supported through expert review, while face validity was confirmed during the pilot test, where feedback from participants led to the refinement of wording for better comprehension. These steps collectively ensured that the instrument was contextually appropriate for assessing awareness, attitudes, and barriers to breast cancer screening among Saudi women. The research focused on the participants′ awareness, attitudes, and perceived obstacles to breast cancer screening as dependent variables, while independent variables encompassed sociodemographic characteristics and factors that could influence awareness and attitudes.

### 2.5. Statistical Analysis Plan

Data was coded and analyzed using SPSS software Version 23.0. Descriptive statistics were applied to present demographic variables. Normality of the data was tested using the Kolmogorov‐Smirnov test, along with histogram and *Q*–*Q* plots. Qualitative data was expressed as numbers and percentages, and quantitative data was presented as means and standard deviations or medians and interquartile ranges, as appropriate. To calculate the awareness score, a common grading method was used and applied to awareness questions where a correct answer was given 1 point and incorrect or neutral answers were given 0 point. A participant who correctly answered more than 50% of the awareness questions (i.e., scored 12 or more out of 23 points) was considered to have a good level of awareness about breast cancer screening. For attitude questions, the answers were graded by giving a score from 3 to 0; a participant who scored 5 or more out of 8 points was considered to have a positive attitude about breast cancer screening. The > 50% thresholds (12 out of 23 for knowledge and 5 out of 8 for attitude) were based on the midpoint principle, which is a commonly used method in KAP studies when validated cut‐offs are unavailable, and this has been used in several breast cancer screening researches [20, 21]. Regarding neutral responses in the knowledge questions, we followed the commonly used method in KAP research of scoring both incorrect and neutral answers as 0, as neither reflects correct knowledge, and this has been used in several breast cancer screening research studies [22]. This method reduces the overrepresentation of knowledge levels. In the scope of breast cancer screening, the absence of correct information may be a significant obstacle to screening, as misinformation. When 20% or more of the cells had an expected count of less than 5, Fisher′s exact test was used instead. A multiple logistic regression was used to determine independent significant predictors of awareness and attitude. Odds ratios (ORs) with 95% confidence intervals (CIs) were expressed relative to a reference baseline category. *p* values less than 0.05 were considered statistically significant.

The missing responses were minimal (18 out of 277 participants for only two questions) and revealed no systematic pattern. Given this low proportion and the negligible risk of bias, we used available‐case analysis (pairwise deletion) without imputation, as recommended by Little and Rubin [23].

## 3. Results

The study included 277 women who participated in the breast cancer screening activity week, with a median age of 23 years (IQR: 21–34), ranging from 18 to 60. The most represented age group was 18–25 years (56.7%). The majority of participants were Saudi nationals (92.1%), with about two‐thirds being single (65%). In terms of education level, most participants (73.3%) had completed university studies, while 15.9% had secondary education. More than half were employed (53.1%), 38.6% were not working, and 8.3% were housewives. Smoking was uncommon, with only 5.1% being current smokers and 0.7% being former smokers. Additionally, 14.4% of participants reported a family history of breast cancer (Table 1).

**Table 1:** Sociodemographic characteristics of the study participants (*n* = 277).

**Variable**	**Categories**	**N** **(%)**
Nationality	Saudi	255 (92.1)
	Non‐Saudi	22 (7.9)

Age (in years)	18–25	157 (56.7)
	26–35	57 (20.6)
	36–45	46 (16.6)
	46 or more	17 (6.1)

Marital status	Single	180 (65)
	Married	87 (31.4)
	Divorced	10 (3.6)

Highest educational level	Primary	2 (0.7)
	Intermediate	3 (1.1)
	Secondary	44 (15.9)
	University	203 (73.3)
	Postgraduate	25 (9)

Occupation	Housewife	23 (8.3)
	Working	147 (53.1)
	Not working	107 (38.6)

Smoking	Yes	14 (5.1)
	No	261 (94.2)
	Ex smoker	2 (0.7)

Family history of breast cancer	Yes	40 (14.4)
	No	219 (79.1)
	Do not know	18 (6.5)

*Note:* %, percentage.

Abbreviation: *N*, number.

About awareness of breast cancer screening, 200 participants (72.2%) had heard of screening methods, while 77 (27.8%) had not. The family history of breast cancer was the most recognized risk factor (76.9%), followed by smoking (72.2%), age over 35 years (62.8%), and lack of breastfeeding (53.8%). Other risk factors were less commonly identified, including lack of physical activity (48%), obesity (44%), oral contraceptive use (38.3%), late menopause (36.5%), and first pregnancy after age 30 (31.4%).

Awareness of breast cancer symptoms varied, with a change in breast size being the most recognized symptom (74.4%), followed by a nonpainful breast lump (74%), breast color change (71.8%), and nipple discharge (71.1%). Axillary lymph node enlargement and severe weight loss were the least identified symptoms, recognized by 64.6% and 41.5% of participants, respectively (Table 2).

**Table 2:** Awareness about breast cancer screening among breast cancer screening health education week attendees.

**Variable**	**Yes**	**No**	**Do not know**
	**N** **(%)**		
Have you heard of breast cancer screening methods?	200 (72.2)	77 (27.8)	**—**
Are you aware that the following are risk factors for breast cancer?			
Age more than 35 years	174 (62.8)	53 (19.1)	50 (18.1)
First pregnancy after 30 years age	87 (31.4)	89 (32.1)	101 (36.5)
Late menopause	101 (36.5)	82 (29.6)	94 (33.9)
Women who do not beast feed	149 (53.8)	55 (19.9)	73 (26.4)
Obesity	122 (44)	67 (24.2)	88 (31.8)
Smoking	200 (72.2)	33 (11.9)	44 (15.9)
Family history of breast cancer	213 (76.9)	30 (10.8)	34 (12.3)
Oral contraceptive pill	106 (38.3)	73 (26.4)	98 (35.4)
Lack of physical activity	133 (48)	58 (20.9)	86 (31)
Are you aware that the following are the symptoms for breast cancer?			
Nonpainful lump in the breast	205 (74)	34 (12.3)	38 (13.7)
Change in breast color	199 (71.8)	31 (11.2)	47 (17)
Nipple discharge	197 (71.1)	31 (11.2)	49 (17.7)
Severe weight loss	115 (41.5)	55 (19.9)	107 (38.6)
Axillary lymph node enlargement	179 (64.6)	40 (14.4)	58 (20.9)
Change in size of the breast	206 (74.4)	28 (10.1)	43 (15.5)

*Note:* %, percentage.

Abbreviation: *N*, number.

**Figure 1:**
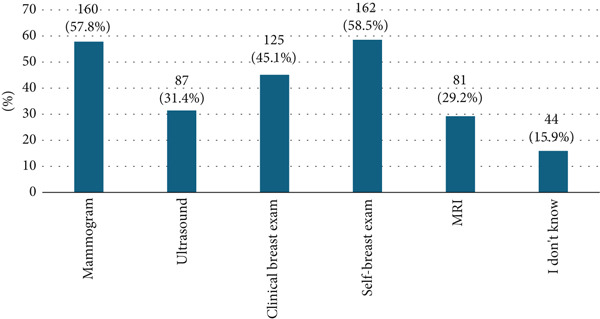
Participants′ awareness about breast cancer screening methods.

For breast cancer screening methods, self‐breast examination was the most commonly identified, mentioned by 162 participants (58.5%), followed by mammography (57.8%) and CBE (45.1%). Ultrasound and MRI were recognized by fewer participants, with 87 (31.4%) and 81 (29.2%) reporting these methods, respectively (Figure 1).

**Figure 2:**
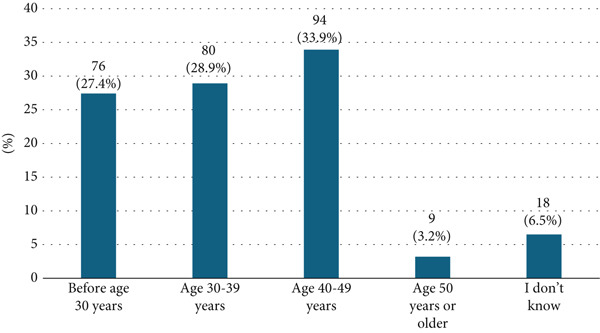
Participants′ awareness about the age of breast cancer screening.

Regarding the recommended starting age for breast cancer screening, only a third of the participants (33.9%) correctly identified the 40–49 age range. In contrast, 80 participants (28.9%) believed screening should start between 30 and 39 years, while 76 (27.4%) thought it should begin before age 30 (Figure 2).

**Figure 3:**
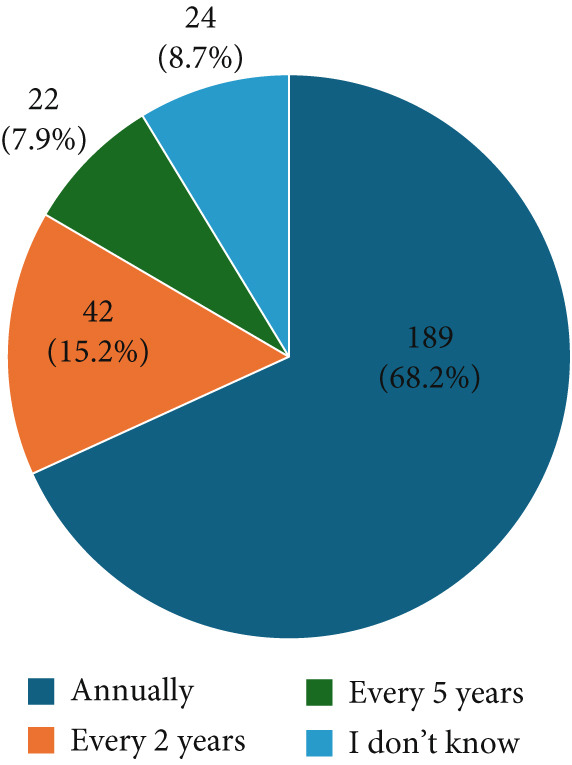
Participants′ awareness about the number of periodic breast cancer screening.

When asked about the recommended frequency of breast cancer screening, over two‐thirds of participants (68.2%) correctly indicated that it should be done annually, while 42 participants (15.2%) believed it should be conducted every 2 years (Figure 3).

Most participants (*n* = 255, 92.1%) regarded breast cancer screening as very important for early detection and treatment, while 22 (7.9%) viewed it as somewhat important. Comfort levels with screening varied, with 129 participants (46.6%) feeling very comfortable and about a third (33.6%) somewhat comfortable during the process. A strong majority (89.2%) believed that screening aids in early detection, improving outcomes. Reported barriers to regular screening included lack of time (30.3%), limited access to screening services (15.2%), fear of the procedure (15.2%), perceived lack of necessity (11.9%), and fear of the potential results (9%) (Table 3).

**Table 3:** Attitude and barriers to breast cancer screening.

**Variable**	**Categories**	**N** **(%)**
How important do you think breast cancer screening is for early detection and treatment	Very important	255 (92.1)
	Somewhat important	22 (7.9)
	Not very important	0 (0)
	Not important at all	0 (0)

How comfortable are you with undergoing breast cancer screening	Very comfortable	129 (46.6)
	Somewhat comfortable	93 (33.6)
	Not very comfortable	40 (14.4)
	Not comfortable at all	15 (5.4)

Do you believe that breast cancer screening can help to detect the cancer early and thus improve the outcome of the disease	Yes	247 (89.2)
	No	5 (1.8)
	Do not know	7 (2.5)
	Missing	18 (6.5)

What are the main reasons you might avoid breast cancer screening?	Being terrified of the possible result	25 (9)
	Cultural believes	6 (2.2)
	Difficulty of getting access to screening services	42 (15.2)
	Fear of the procedure	42 (15.2)
	Lack of female doctors	5 (1.8)
	Lack of information about its importance	18 (6.5)
	Lack of time	84 (30.3)
	Perceived lack of necessity	33 (11.9)
	Other	4 (1.4)
	Missing	18 (6.5)

*Note:* %, percentage.

Abbreviation: *N*, number.

The mean overall awareness score among participants was 12.6 ± 5.5 (range: 0–22) out of 23. A majority, 176 participants (63.5%), demonstrated a good level of awareness about breast cancer screening, while 101 (36.5%) had a poor level of awareness. The median attitude score was 7 (IQR: 6–8); nearly all participants (97.8%) held a positive attitude toward breast cancer screening, while a small fraction (2.2%) held a negative attitude.

An analysis of the association between breast cancer screening awareness and participants′ sociodemographic characteristics revealed significant relationships with age and occupation (*p* = 0.003 and 0.001, respectively). Participants aged 46 and older, as well as those who were employed, exhibited higher awareness levels. Other factors, such as nationality, marital status, education, smoking status, and family history, showed no significant association with awareness levels (*p* > 0.05).

Similarly, examining the association between attitude toward breast cancer screening and sociodemographic factors indicated that education level had a significant relationship with attitude (*p* = 0.045). Participants with postgraduate education displayed the most positive attitudes toward screening. Other factors, including nationality, age, marital status, occupation, smoking, and family history, were not significantly associated with attitude toward screening (*p* > 0.05) (Table 4).

**Table 4:** Association between awareness and attitudes toward breast cancer screening with sociodemographic characteristics.

**Variable**	**Awareness**		**Odds ratio (95% CI)**	**p** **value**	**Attitude**		**Odds ratio (95% CI)**	**p** **value**
	**Good**	**Poor**			**Positive**	**Negative**		
	**N** **(%)**				**N** **(%)**			
Nationality:								
Saudi	160 (62.7)	95 (37.3)	1	0.351^a^	249 (97.6)	6 (2.4)	1	1.000^b^
Non‐Saudi	16 (72.7)	6 (27.3)	1.583 (0.599–4.185)		22 (100)	0 (0)	3.89 × 10^7^ (0.00)	
Age (in years):								
18–25	95 (60.5)	62 (39.5)	1	0.003^a^^∗^	152 (96.8)	5 (3.2)	1	0.502^b^
26–35	40 (70.2)	17 (29.8)	1.536 (0.800–2.946)		57 (100)	0 (0)	5.314 × 10^7^ (0.00)	
36–45	24 (52.2)	22 (47.8)	0.712 (0.368–1.379)		45 (97.8)	1 (2.2)	1.48 (0.169–12.99)	
46 or more	17 (100)	0 (0)	1.05 × 10^9^ (0.000)		17 (100)	0 (0)	5.314 × 10^7^ (0.00)	
Marital status:								
Single	108 (60)	72 (40)	1	0.249^a^	175 (97.2)	5 (2.8)	1	0.734^b^
Married	61 (70.1)	26 (29.9)	1.564 (0.905–2.704)		86 (98.9)	1 (1.1)	2.45 (0.28–1.359)	
Divorced	7 (70)	3 (30)	1.556 (0.389–6.214)		10 (100)	0 (0)	4.616 × 10^7^ (0.00)	
Education:								
Primary	1 (50)	1 (50)	1	0.370^b^	1 (50)	1 (50)	1	0.045^b^^∗^
Intermediate	2 (66.7)	1 (33.3)	2.00 (0.051–78.250)		3 (100)	0 (0)	1.615 × 10^9^ (0.00)	
Secondary	23 (52.3)	21 (47.7)	1.095 (0.064–18.63)		43 (97.7)	1 (2.3)	43 (1.424–1298.66)	
University	131 (64.5)	72 (35.5)	1.819 (0.112–29.52)		199 (98)	4 (2)	49.75 (2.62–944.1)	
Postgraduate	19 (76)	6 (24)	3.167 (0.17–58.703)		25 (100)	0 (0)	1.615 × 10^9^ (0.00)	
Occupation:								
Housewife	14 (60.9)	9 (39.1)	1	0.001^a^^∗^	23 (100)	0 (0)	1	0.886^b^
Working	108 (73.5)	39 (26.5)	1.780 (0.714–4.440)		144 (98)	3 (2)	0.000 (0.000)	
Not working	54 (50.5)	53 (49.5)	0.655 (0.261–1.642)		104 (97.2)	3 (2.8)	0.000 (0.000)	
Smoking:								
Yes	8 (57.1)	6 (42.9)	1	0.896^b^	14 (100)	0 (0)	1	1.000^b^
No	167 (64)	94 (36)	1.332 (0.449–3.956)		255 (97.7)	6 (2.3)	0.000 (0.000)	
Ex smoker	1 (50)	1 (50)	0.750 (0.039–14.57)		2 (100)	0 (0)	1.000 (0.000)	
Family history of breast cancer:								
Yes	29 (72.5)	11 (27.5)	1	0.373^a^	39 (97.5)	1 (2.5)	1	0.730^b^
No	137 (62.6)	82 (37.4)	0.634 (0.301–1.336)		215 (98.2)	4 (1.8)	1.37 (0.150–12.66)	
Do not know	10 (55.6)	8 (44.4)	0.474 (0.149–1.513)		17 (94.4)	1 (5.6)	0.436 (0.026–7.38)	

*Note:* %, percentage.

Abbreviations: CI, confidence interval; *N*, number.

^a^Chi‐square test.

^b^Fisher′s exact test.

^∗^Significant *p* value.

The multiple logistic regression analysis was applied to assess predictors of awareness and attitude toward breast cancer screening. The results revealed that none of the demographic variables were significant predictors of having good awareness of breast cancer screening after adjusting for all other factors in the model (*p* > 0.05). Similarly, none of the demographic variables were significant predictors of having a positive attitude toward breast cancer screening after adjusting for all other factors in the model (*p* > 0.05) (Tables 5 and 6).

**Table 5:** Predictors of awareness of breast cancer screening.

**Variable**	**Odds ratio**	**95% CI for odds ratio**		**p** **value**
		**Lower**	**Upper**	
Nationality:				
Saudi	1			
Non‐Saudi	1.542	0.464	5.119	0.479
Age (in years):				
18–25	1			
26–35	0.477	0.169	1.346	0.162
36–45	0.175	0.059	0.519	0.002
46 or more	2.33 × 10^16^	0.000	—	0.997
Marital status:				0.475
Single	1			
Married	1.693	0.715	4.010	0.231
Divorced	1.117	0.186	6.716	0.904
Education:				0.657
Primary	1			
Intermediate	1.815	0.000	—	1.000
Secondary	6.062 × 10^8^	0.000	—	0.998
University	1.045 × 10^9^	0.000	—	0.998
Postgraduate	1.309 × 10^9^	0.000	—	0.998
Occupation:				0.001
Housewife	1			
Working	1.206	0.409	3.553	0.735
Not working	0.296	0.089	0.986	0.047
Smoking:				0.704
Yes	1			
No	1.545	0.429	5.565	0.506
Ex smoker	0.702	0.028	17.697	0.830
Family history of breast cancer:				0.287
Yes	1			
No	0.610	0.267	1.393	0.240
Do not know	0.365	0.101	1.327	0.126

Abbreviation: CI, confidence interval.

**Table 6:** Predictors of attitude toward breast cancer screening.

**Variable**	**Odds ratio**	**95% CI for odds ratio**		**p** **value**
		**Lower**	**Upper**	
Nationality:				
Saudi	1			
Non‐Saudi	5.213 × 10^6^	0.000	—	0.998
Age (in years):				0.721
18–25	1			
26–35	4.047 × 10^6^	0.000	—	0.997
36–45	0.179	0.010	3.323	0.248
46 or more	1.318 × 10^8^	0.000	—	0.999
Marital status:				1.000
Single	1			
Married	26.332 × 10^6^	0.000	—	0.996
Divorced	4.389	0.000	—	1.000
Education:				1.000
Primary	1			
Intermediate	11.546 × 10^8^	0.000	—	0.999
Secondary	8.208 × 10^13^	0.000	—	0.997
University	10.052 × 10^13^	0.000	—	0.997
Postgraduate	11.622 × 10^20^	0.000	—	0.996
Occupation:				0.969
Housewife	1			
Working	0.000	0.000	—	0.999
Not working	0.000	0.000	—	0.999
Smoking:				1.000
Yes	1			
No	0.000	0.000	—	0.999
Ex smoker	6.191	0.000	—	1.000
Family history of breast cancer:				0.548
Yes	1			
No	1.810	0.152	21.512	0.638
Do not know	0.487	0.023	10.403	0.645

Abbreviation: CI, confidence interval.

## 4. Discussion

### 4.1. Demographic Insights

Our study revealed that most participants were young adults (mean age 28.1 years, majority 18–25 years) and predominantly college graduates (73.3%). This demographic profile suggests that the findings primarily reflect the perspectives of younger, educated Saudi women rather than the wider female population. Similar patterns have been observed in recent Saudi studies, where higher education was associated with greater awareness and health‐seeking behaviors [24]. However, reliance on younger age groups may underestimate the knowledge gaps and barriers faced by older or less educated women, who are typically at greater risk for breast cancer. The relatively high percentage of college graduates (73.3%) in our sample is notable since the increased education level has been positively associated with greater awareness and positive health behaviors, as shown by Al‐Zalabani et al. [25], therefore highlighting the potential utility of educational interventions within this population cohort.

### 4.2. Knowledge and Awareness

Awareness of breast cancer screening methods was relatively high (72.2%), consistent with findings from Al‐Zalabani et al. [25] and more recent reports showing improvements in knowledge among Saudi women [8]. Participants most frequently recognized self‐breast examination (58.5%) and mammography (57.8%). Yet, awareness of more advanced modalities such as MRI (29.2%) and ultrasound (31.4%) was limited, echoing global disparities in awareness between low‐ and high‐resource settings [26]. Although family history (76.9%) and smoking (72.2%) were commonly identified as risk factors, fewer participants recognized obesity (44%) or late menopause (36.5%), despite strong epidemiological evidence linking these with breast cancer risk [27]. Lastly, the family history of breast cancer was reported by 14.4% of our sample participants, which reflects the proportion of the global risk factors related to familial breast cancer in the general population [14]. Awareness of smoking as a risk factor aligns with several studies, which reported a strong understanding of lifestyle‐related risks among women in urban settings [8, 28]. These gaps suggest that public health campaigns should address overlooked lifestyle and reproductive risk factors. Awareness levels in developed countries such as the United States are reportedly higher, with 80%–90% of women being informed about screening methods [29]. These differences may be due to the availability of resources and the frequency of public health campaigns.

### 4.3. Attitudes Toward Screening

The breast cancer screening method most commonly recognized by participants was the self‐breast exam (58.5%), followed closely by mammography (57.8%) and clinical breast exams (45.1%). These results are consistent with research conducted in Saudi Arabia, including studies by Abdel‐Salam et al. [18], which indicated a high level of awareness regarding self‐breast exams and mammograms among women participating in health education initiatives [18].

The vast majority (92.1%) of participants expressed positive attitudes toward screening, acknowledging its value for early detection and improved outcomes. This optimism aligns with global trends indicating growing acceptance of screening as a preventive practice [30]. However, nearly one‐third of women recommended screening initiation before the age of 30, which deviates from current Saudi Ministry of Health guidelines recommending mammography for women aged 40–69 [31, 32]. This discrepancy reflects uncertainty influenced by family history, social media, and regional variations in health communication and suggests the need for clearer, consistent messaging.

### 4.4. Barriers and Challenges

Despite positive attitudes, several barriers were reported, including lack of time (30.3%), difficulties accessing services (15.2%), and fear of procedures (15.2%). These findings mirror barriers identified in Middle Eastern and African contexts, where logistical and psychosocial challenges hinder uptake [33, 34]. According to the Health Belief Model (HBM), these represent perceived barriers that reduce screening intention despite high awareness and positive attitudes. Furthermore, a positive attitude was prevalent, with an average attitude score of 7.0 ± 1.0 out of 8. A significant majority (97.8%) expressed a positive attitude toward breast cancer screening, consistent with research by Abdel‐Salam et al. [18] indicating high positivity toward screening among Saudi women [18]. Addressing such barriers through mobile screening units, flexible appointment systems, and culturally sensitive counseling could enhance participation.

### 4.5. Broader Social and Cultural Context

Breast cancer screening in Saudi Arabia cannot be fully understood without considering the broader sociocultural environment. Family support, particularly from male relatives, often influences women′s healthcare decisions [35]. Integrating family‐centered education, mosque‐based awareness initiatives, and community leader engagement may enhance screening uptake. Moreover, regional disparities within Saudi Arabia—such as urban versus rural differences—warrant tailored awareness strategies.

### 4.6. Technological and Innovative Approaches

Digital health technologies present new opportunities to address screening barriers. Mobile applications that provide reminders for self‐breast examinations, telehealth consultations to reduce stigma, and social media campaigns targeting younger women could play a vital role. Recent studies in Saudi Arabia demonstrate growing public receptiveness to mHealth tools for cancer prevention [24]. Incorporating these approaches into national screening campaigns could improve accessibility, especially for younger, tech‐savvy populations.

### 4.7. Strengths and Limitations

This study contributes valuable insights into community awareness, attitudes, and perceived barriers toward breast cancer screening in the Saudi context. One of its strengths lies in the use of a predesigned and pretested questionnaire adapted from earlier validated studies, combined with expert review and pilot testing to ensure clarity and cultural appropriateness. Collecting data during a health education campaign also allowed access to a diverse sample of women who were directly engaged in screening‐related activities, thereby enhancing the practical relevance of the findings.

However, certain limitations should be acknowledged. First, the timing and context of data collection—during a health education campaign—may have influenced participants′ awareness and attitudes, potentially introducing contextual bias and leading to an overestimation of knowledge and positive perceptions. Recent methodological reviews confirm that research settings and heightened awareness interventions can shape participants′ responses, sometimes leading to inflated outcomes [36]. Second, while the questionnaire was piloted and demonstrated acceptable reliability, formal psychometric testing, such as Cronbach′s alpha or factor analysis, was not conducted. This limits the robustness of the tool′s reliability and construct validity. Current best practice guidelines emphasize the importance of conducting full psychometric validation, including reliability and construct validity testing, particularly when instruments are adapted for different cultural contexts [37]. Future research should aim to collect data outside campaign settings to minimize contextual influence and should undertake comprehensive psychometric validation to strengthen confidence in the reliability and generalizability of findings. Third, the use of a convenience sampling method may restrict the generalizability of the findings due to potential selection bias, as the sample may not adequately represent all women in Riyadh or across Saudi Arabia. However, despite this limitation, the targeted approach allowed for a more focused and impactful dissemination of information on breast cancer awareness and early detection methods. However, in future studies, we will consider probabilistic sampling methods to strengthen representativeness. Data collection relied on self‐reported responses, which are susceptible to recall and social desirability biases, potentially compromising the accuracy of reported awareness, attitudes, and barriers. Additionally, stratified analyses by subgroups such as age or education were not conducted due to sample size constraints, which may have restricted the identification of nuanced patterns. Finally, the study′s cross‐sectional design provides a snapshot of awareness and attitudes at a specific point in time but does not facilitate an assessment of changes over time or causality between variables.

## 5. Conclusion

Findings demonstrate a relatively high level of awareness and favorable attitudes toward breast cancer screening among attendees at KSMC′s breast cancer screening health education week in Riyadh. However, significant barriers, such as time constraints, access challenges, and screening‐related fears, continue to deter regular screening participation. Future initiatives should aim to address these barriers by implementing targeted awareness campaigns that clarify screening intervals and reduce fear associated with screening. Improving access, possibly through mobile clinics or extended service hours, could enhance participation rates. Continuous education and encouragement from healthcare providers will be essential to improve adherence to screening guidelines, ultimately aiding in early detection and reducing breast cancer mortality in Saudi Arabia.

## 6. Practical Implications and Policy Relevance

The findings highlight opportunities to strengthen breast cancer awareness and screening uptake in alignment with Saudi Vision 2030 health priorities, which emphasize preventive healthcare and women′s health empowerment [32]. Practical recommendations include the following:

• Expanding targeted awareness campaigns addressing lifestyle‐related risk factors.
• Clarifying national screening guidelines to reduce misinformation on screening age and frequency.
• Leveraging digital health platforms and mobile units to overcome logistical barriers.
• Incorporating family and community leaders into awareness strategies to address cultural influences.

By integrating educational, technological, and policy‐driven interventions, these measures could enhance breast cancer screening uptake and contribute to reducing morbidity and mortality among Saudi women.

## Ethics Statement

The study was conducted after the approval of the Institutional Review Board of King Saud Medical City, Riyadh, Research and Innovation Center (Protocol Code H1RI‐01‐Oct24‐01, and the date of approval was October 15, 2024).

## Conflicts of Interest

The authors declare no conflicts of interest.

## Funding

No funding was received for this manuscript.

## Data Availability

The data that support the findings of this study are available on request from the corresponding author.
